# A cluster randomised control trial to evaluate the effectiveness and cost-effectiveness of the Italian medicines use review (I-MUR) for asthma patients

**DOI:** 10.1186/s12913-017-2245-9

**Published:** 2017-04-24

**Authors:** Andrea Manfrin, Michela Tinelli, Trudy Thomas, Janet Krska

**Affiliations:** 1 Medway School of Pharmacy, Universities of Greenwich and Kent at Medway, Anson Building, Central Avenue, Chatham Maritime, Chatham, Kent ME4 4TB UK; 2 LSE Health and Social Care, The London School of Economics and Political Science, Houghton Street, London, WC2A 2AE UK

**Keywords:** Asthma control, Medicines use review, Community pharmacy, Effectiveness, Cost-effectiveness

## Abstract

**Background:**

The economic burden of asthma, which relates to the degree of control, is €5 billion annually in Italy. Pharmacists could help improve asthma control, reducing this burden. This study aimed to evaluate the effectiveness and cost-effectiveness of Medicines Use Reviews provided by community pharmacists in asthma.

**Methods:**

This cluster randomised, multi-centre, controlled trial in adult patients with asthma was conducted in 15 of the 20 regions of Italy between September 2014 and July 2015. After stratification by region, community pharmacists were randomly allocated to group A (trained in and delivered the intervention at baseline) or B (training and delivery 3 months later), using computerised random number generation in blocks of 10. Each recruited up to five patients, with both groups followed for 9 months.

The intervention consisted of a systematic, structured face-to-face consultation with a pharmacist, covering asthma symptoms, medicines used, attitude towards medicines and adherence, recording pharmacist-identified pharmaceutical care issues (PCIs). The primary outcome was asthma control, assessed using the Asthma-Control-Test (ACT) score (ACT ≥ 20 represents good control). Secondary outcomes were: number of active ingredients, adherence, cost-effectiveness compared with usual care. Although blinding was not possible for either pharmacists or patients, assessment of outcomes was conducted by researchers blind to group allocation.

**Results:**

Numbers of pharmacists and patients enrolled were 283 (A = 136; B = 147) and 1263 (A = 600; B = 663), numbers completing were 201 (A = 97; B = 104) and 816 (A = 400; B = 416), respectively. Patients were similar in age and gender and 56.13% (458/816) had poor/partial asthma control. Pharmacists identified 1256 PCIs (mean 1.54/patient), mostly need for education, monitoring and potentially ineffective therapy. Median ACT score at baseline differed between groups (A = 19, B = 18; *p* < 0.01). Odds ratio for improved asthma control was 1.76 (95% CI 1.33–2.33) and number needed to treat 10 (95% CI 6–28). Number of active ingredients reduced by 7.9% post-intervention (*p* < 0.01). Adherence improved by 35.4% 3 months post-intervention and 40.0% at 6 months (*p* < 0.01). The probability of the intervention being more cost-effective than usual care was 100% at 9 months.

**Conclusions:**

This community pharmacist-based intervention demonstrated both effectiveness and cost-effectiveness. It has since been implemented as the first community pharmacy cognitive service in Italy.

**Trial registration:**

TRN: ISRCTN72438848 (registered 5^th^ January 2015, retrospectively).

## Background

The prevalence of asthma has been increasing since the late 1990’s and it has been estimated that about 400 million people will suffer from asthma by 2025 [1, 2]. Currently the number of disability-adjusted life years (DALYs) lost to lack of control of asthma worldwide translates into a global loss of 15 million DALYs per year and an estimated one in every 250 deaths worldwide is caused by asthma. Asthma accounts for an economic loss of €72 billion annually in the 28 countries of the EU [3]; this includes the annual costs of health care (about €20 billion), the loss of productivity for patients (€14 billion), and a monetised value of DALYs lost of €38 billion. In the UK the burden of asthma was estimated at a cost to the NHS of more than £1 billion/year, including prescribing, hospital admissions and primary care consultations [4, 5]. In Italy the latest estimate showed an asthma prevalence of 6.2% [6] with an economic loss of €5 billion annually_._ An Italian cost of illness study, published in 2000, reported a mean annual cost for an asthmatic patient of €741 (95% CI: 599–884), ranging from €379 (95% CI: 216–541) for well-controlled asthmatics to €1,341 (95% CI: 978–1,706) for poorly controlled cases, the latter accounting for 46.2% of the total cost of asthma [7]. In 2006 the Global Initiative for Asthma (GINA) advocated a new asthma management approach, based on asthma control rather than asthma severity [8], since “the principal objective of asthma treatment is to achieve asthma control”. The GINA 2015 pocket guide for asthma management and prevention [9] defines asthma control as the extent to which the effects of asthma can be seen in patients, or has been reduced or removed by treatment. While guidance on asthma control abounds in the UK [10, 11] and other countries, [12] many patients do not achieve good asthma control, with negative implications for their health and quality of life, as well as increased health care costs and loss of productivity [6, 13, 14]. Asthma control may be affected by co-morbidity, continued exposure to triggers, behavioural issues, patient preferences, ineffective treatment, poor adherence and costs for patients [15].

Pharmaceutical care, defined in Europe [16] as “‘the pharmacists’ contribution to the care of individuals in order to optimise medicines use and improve health outcomes”, could contribute to asthma management. Many studies report positive effects of pharmacist interventions in asthma [17–28]. Indeed, a systematic review published in 2016 [29] looking at the impact of pharmacists’ intervention on clinical outcomes in asthma identified 21 studies. However of these, only nine adopted a randomised control or a cluster randomised control design, only ten measured asthma control as main outcome as suggested by GINA management approach in assessing effectiveness, [9] and only one included an economic evaluation of the pharmacy intervention [22]. Pharmacy-led approaches to asthma management include both one-off interventions, such as Medicines Use Review (MUR), and longer-term provision of pharmaceutical care. In England, the MUR is a cognitive pharmaceutical service, funded by the government, [30] where accredited pharmacists can undertake structured adherence-centred reviews with patients using multiple medicines, particularly those receiving medicines for asthma and other long-term conditions. Similar services exist in some European (Denmark, Switzerland) and other countries (Australia, Canada, New Zealand, USA) [31], whereas in Italy such services (for asthma or other conditions) have not been introduced. Therefore, no empirical research exists addressing the effectiveness or cost-effectiveness of a MUR service in Italy.

This study describes the first attempt to deliver a novel community pharmacy intervention for asthma patients, adapted from the MUR service for chronic diseases in England, across the Italian territory and to evaluate its effectiveness and cost-effectiveness. The key research questions were:

Is the Italian Medicines Use Review (I-MUR) service provided by community pharmacists:

i. Effective in
  - Improving asthma control as assessed by the Asthma Control Test (ACT) score?
  - Optimising the number of medicines (active ingredients) used by patients?
  - Identifying pharmaceutical care issues?
  - Improving patients’ adherence to asthma medications?
ii. Cost-effective for the healthcare system and the society (compared with usual care) in terms of cost per quality adjusted life year gained?

## Methods

The study was a cluster randomised controlled trial, with phased intervention, as defined by Gums et al. (2016) [32], in order to avoid selection bias, with control clusters providing the intervention after the primary end point (primary outcome) was finished (T3). Therefore all pharmacists (intervention and control) provided the I-MUR service to patients with asthma, but at different time points during the study. The study protocol is published elsewhere [33].

Community pharmacists were recruited from 15 out of the 20 Italian regions: Trentino Alto Adige, Lombardia, Sicilia, Puglia, Sardegna, Piemonte, Valle d’Aosta, Veneto, Friuli Venezia Giulia, Toscana, Emilia Romagna, Marche, Abruzzo, Lazio and Campania. It was conducted between September 2014 and July 2015 with data collection at 3-months intervals (at baseline (T0); at 3 months (T3); at 6 months (T6); at 9 months (T9)). Pharmacists were randomly allocated to two groups after stratification by region: group A were trained to provide the I-MUR immediately after completion of the baseline ACT score at T0; group B received training and provided this service 3 months later, at T3.

### Intervention

The I-MUR consisted of a systematic, structured interview, conducted in a private room within the pharmacy, which covered asthma symptoms, medicines used, attitudes towards medicines and adherence. The pharmacists were trained to identify pharmaceutical care issues (PCIs) which could impact on optimal medicines use or asthma control and provide advice to the patients and recommendations to their GP, as necessary. As a process measure, the number of PCIs identified by pharmacists during the I-MUR service provision, classified using the method of Krska et al [34], were recorded.

### Outcome measures

Primary outcome measure: Asthma control at baseline (T0) and at 3-months intervals (T3, T6 and T9), assessed using the ACT score. This measure has previously been used in Italy, in a study conducted by the Italian Society of General Medicine (Societá Italiana di Medicina Generale) [35]. It was defined by international guidelines as: ACT ≤ 14 = not controlled, 15 ≤ ACT ≤ 19 = partially controlled, and ACT ≥ 20 controlled [36]. A clinically relevant difference was defined as change from not controlled/partially controlled to controlled asthma.

Secondary outcome measures were:

1. Number of active ingredients used by patients at the point of delivery of the I-MUR provision and 3-months follow-up, as reported by patients.
2. Patients’ self-reported adherence to asthma medication at the point of delivery of the I-MUR provision and 3-months follow-up, measured using two questions adapted from the Morisky Medication-Adherence Scale-MMAS (8-item) [37] embedded within the I-MUR interview.
3. Cost-effectiveness of I-MUR asthma service compared with usual care, measured in terms of cost per QALY (as a measure of disease burden, including both the quality and the quantity of life) gained, at different time points.

### Recruitment, inclusion criteria, randomization and blinding

The study was conducted with the support of the Italian Pharmacists’ Federation (FOFI), which supported pharmacist recruitment in each region. Eligible pharmacists, working in pharmacies with private consultation facilities and internet connection, were identified by FOFI and invited to participate in the study. Those who agreed received training in providing the I-MUR, patient recruitment and data collection by AM. Each pharmacist was instructed to recruit five adult patients with an asthma diagnosis or at least 6 months consecutive use of medicines indicating asthma.

Pharmacists within each region were randomly allocated to Group A (intervention) or B (control) by JK, using computerised random number generation in blocks of 10. AM provided training at either T0 (Group A) or T3 (Group B), thus neither the research team nor the pharmacists were blind to delivery of the intervention. All data, comprising both primary and secondary outcome measures, were collected by the pharmacists and entered anonymously onto a web-based template, to which the researchers remained blind until an interim analysis took place at T6, which was required by the funders.

### Sample size, training and study-timeline

The study aimed to involve 360 pharmacists and 1800 patients, with each pharmacist recruiting five patients. Full inclusion and exclusion criteria for both pharmacists and patients are described in the trial protocol, [33] along with details of the training provided to pharmacists and the study timeline.

Neither the pharmacist nor patient numbers anticipated were achieved, therefore it was necessary to determine the post-hoc power of the study. In the light of the number of pharmacists and patients enrolled and remaining in the study from T0 to T9, (see results), the actual power of the study was calculated maintaining the intra-cluster correlation coefficient (ICC) value of 0.02 and level of significance (two-tailed alpha) at 0.05 as originally proposed (SPSS Sample Power 3, IBM) [38].

### Data sources

ACT score was obtained at all four time points, adherence and number of active ingredients on three occasions and PCIs once; more detail is available in the protocol.

Due to budget constraints primary data on cost and utility outcomes were not collected. Evidence on direct and indirect costs [6, 13] and health benefit (e.g. utility assessed using EuroQol 5 dimensions, EQ-5D) [39] was extrapolated from the literature and used to inform the economic evaluation (see below).

### Effectiveness analysis

Effectiveness data on asthma control, number of active ingredients used, number of PCIs, patients’ adherence to asthma medication were visually inspected using the quantile-quantile plot and the Kolmogorov-Smirnov test to check for normality. Data were not normally distributed therefore non-parametric techniques were used for analysis. Differences between groups A and B were compared using Mann-Whitney *U* test for continuous variables and *X* ^2^ (Chi square) for categorical variables. Wilcoxon Sign Rank Test was used for continuous variables within the same group at different time points (T0, T3, T6 and T9) [40]. Variation of ACT score across T0, T3, T6 and T9 was conducted using Friedman’s ANOVA, instead of one way repeated measure ANOVA as anticipated in the protocol. Correspondence analysis (CA), a multivariate analysis technique which allows graphical representation of data showing how variables are related, was used to assess the relationship between ACT scores and adherence. The primary outcome (ACT score) was analysed using two separate approaches: intention to treat (ITT) analysis, applying the last observation carried forward (LOCF), and per protocol analysis (PP). A separate analysis of primary outcome at T3, using generalised estimating equations (GEE), was conducted as recommended by Consort Statement 2010 [41] and Galbraith et al. (2010) [42] for cluster data, because GEE is a semiparametric technique used for continuous non-normal, binary and categorical responses. Moreover the Agency for Healthcare Research and Quality (AHRQ) suggests using GEE to either repeated measures at fixed intervals or for a single measure accounting for the clustering effect (multi-site study) as in our case [43]. Binary logistic analysis was used, dichotomising outcomes into not-controlled (ACT ≤ 19) and controlled (ACT ≥ 20). The GEE model was adjusted for the ACT score at baseline using age and gender. The relative risk (RR), the relative risk reduction (RRR), the absolute risk reduction (ARR) and the number needed to treat (NNT) were calculated using the PP results. An online calculator provided by the Centre for Evidence-Based Medicine of Toronto was used to confirm the results calculated manually, which also provided significance levels (p value) and 95% confidence intervals (CI) [44].

Secondary outcomes (number of active ingredients used, number of PCIs, adherence) were analysed only for patients completing the trial (PP). All analyses were conducted using SPSS software v22.

### Cost effectiveness analysis

Published data from European [6, 13] and Italian population-based studies monitoring the annual cost [6] and quality of life [39] in terms of EQ-5D relating to ACT score were used for this analysis. Average annual cost and utility estimates per patient were extrapolated for not controlled, partially controlled, and controlled patients (see ACT score categories above) and linked to the individual patient ACT scores reported at all four time points (T0, T3, T6, and T9). Group B patients were defined as receiving usual care by keeping ACT scores measured at T3 (prior to receiving the I-MUR service) constant across time. A sensitivity analysis was conducted keeping Group B ACT scores at T3 constant across time (T6, T9). As mean ACT score values differed between the groups at baseline, changes in costs and QALY over the follow-up period considering three separate scenarios, (T3, T6 and T9) were analysed.

The primary economic analysis compared I-MUR service and usual care from the public healthcare perspective whereas the secondary analysis considered both direct and indirect costs. For the primary analysis we used Vervloet et al. (2006) [13] cost data on scheduled healthcare visits to their usual physician and specialist, unscheduled healthcare asthma related in-patient admissions, emergency visits, and emergency contacts with a physician. The secondary analysis included indirect costs on productivity losses (working days lost) and leisure time forgone (days with limited, not work-related activities) as well as direct costs on doctor visits, clinical and laboratory tests, pharmacological treatment, emergency visits, and hospital admissions, using data from Accordini et al. (2013) [6]. Costs were actualised to 2015 values using the appropriate consumer price index [45]. The cost for delivering the I-MUR service to Group A at T0 was €40, calculated based on data available in the international literature [31]. The summary cost-effectiveness statistic calculated was the incremental cost-effectiveness ratio (ICER). Uncertainty and variation around the ICER mean are represented by the cost effectiveness acceptability curve (CEAC), obtained by re-sampling the data 1000 times to generate a mean cost and life year or QALY gain from each group, using a nonparametric bootstrap approach. The proportion of re-sampled datasets for which the calculated ICER lies below a given threshold is interpreted as the probability that the ICER of the intervention is below that threshold. Due to the lack of an official willingness-to-pay (WTP) threshold in Italy, we used a threshold of €30,000 (£24,017) per QALY, which is within the values recognized by the National Institute for Health and Care Excellence range of acceptable cost effectiveness (£20,000–30,000 per QALY). Data analysis was performed using STATA v 13.

## Results

**Fig. 1:**
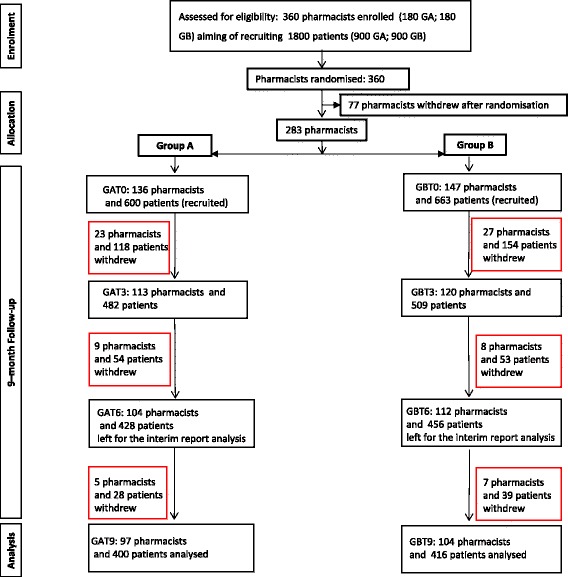
CONSORT diagram describing the flow of participants though the study

There were 360 pharmacists randomised, of whom 283 started the study, recruiting 1263 patients at T0, with 201 pharmacists and 816 patients completing the study and having data at each time point. The median number of patients enrolled by each pharmacist was four. The overall drop-out rate from T0 to T9 was 29% (*n* = 82) for pharmacists and 35% (*n* = 447) for patients. The numbers in each group at T9 were 400 (49% of the total) in group A and 416 (51%) in group B (Fig. 1). Based on a 20% increase in the proportion of patients with controlled asthma (ACT score ≥20), considering the number of individuals per cluster (4), the ICC (0.02), the calculated design effect (DE) was 1.06, which provided an effective sample size of 758 patients, and the power attained by the study was 90%.

### Baseline characteristics

The numbers of pharmacists and patients from the 15 regions differed, but patient numbers recruited to Groups A and B within each region was similar. The highest number of patients was found in Sardinia and the lowest in Lazio-Campania. At T0, age range and gender showed no statistical difference between the two groups (Table 1). Overall there was a majority of female patients in both the PP (58.82%; *n* = 480) and ITT analysis (57.17%; *n* = 722).

**Table 1:** Baseline characteristics and ACT scores

	Recruited			Completed		
	Group A	Group B	*p*	Group A	Group B	*p*-value
Number of patients	600	663		400	416	
Female %**	58.80	55.70	0.26	59.80	57.90	0.60
Age range *n* (%)						
18 to 30**	55 (9.17)	68 (10.26)	0.96	32 (8.00)	43 (10.34)	0.69
31 to 40**	81 (13.50)	89 (13.42)	0.96	58 (14.50)	62 (14.90)	0.69
41 to 50**	117 (19.50)	122 (18.40)	0.96	79 (19.75)	77 (18.51)	0.69
51 to 60**	114 (19.00)	125 (18.85)	0.96	73 (18.25)	80 (19.23)	0.69
61 to 70**	125 (20.83)	136 (20.51)	0.96	82 (20.50)	77 (18.51)	0.69
71 to 80**	83 (13.83)	88 (13.27)	0.96	59 (14.75)	52 (12.50)	0.69
Over 81**	25 (4.17)	35 (5.28)	0.96	17 (4.25)	25 (6.01)	0.69
ACT scores						
Median (IQR)*	19 (14.25–23)	17 (17–21)	<0.01	19 (15–23)	18 (18–24)	<0.01
5–14 not controlled *n*(%)**	150 (25.00)	203 (30.60)	<0.01	91 (22.80)	121 (29.10)	0.01
15–19 partially controlled n(%)**	177 (29.50)	223 (33.60)	<0.01	114 (28.50)	132 (31.60)	0.01
20–25 controlled *n*(%)**	273 (45.50)	237 (35.71)	<0.01	195 (48.70)	163 (39.30)	0.01

* Mann-Whitney *U* test, Median (IQR), *p* < 0.05

**Chi-square test, *p* < 0.05

#### Process measure: pharmaceutical care issues (PCIs)

Pharmacists identified PCIs while providing the I-MUR intervention in 527 (64.58%) patients; 277 (69.25%) of Group A patients and 250 (60.00%) of Group B patients. The total number of PCIs identified was 1256, mean for the total population 1.54 per patient, and among patients with PCIs 2.40. Education required was the most frequent PCI identified in both groups, which together with monitoring issues, discrepancy between dose prescribed and dose used, potentially ineffective therapy and potential adverse reactions accounted for 64.70% of all PCIs identified.

### Primary outcome: asthma control

**Fig. 2:**
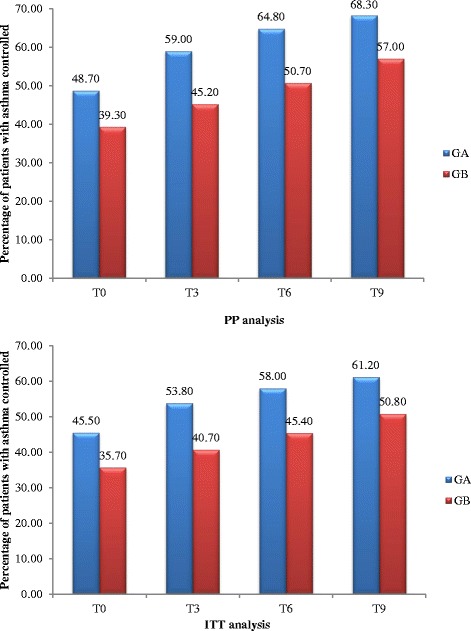
Percentage of patients in group A and B with controlled asthma (ACT score ≥20) at four time points shown as both PP and ITT

Using a per protocol approach to analysis, the overall proportion of patients with poorly controlled asthma at baseline (T0) was 56.13% (458/816). However median ACT score differed significantly between the two groups, being lower in Group B patients, due to a higher proportion having poor asthma control, compared to Group A patients (Table 1). At T3, asthma control in Group A patients, who had received the I-MUR intervention, showed both a statistically significant difference (*p* < 0.01) and a clinically relevant difference compared with baseline, with the median ACT score increasing from 19 (uncontrolled) to 20.50 (controlled) (Table 2). There were 41 of 205 patients (20.00%) with ACT score ≤20 who achieved control, and the proportion of patients with controlled asthma increased from 48.70 to 59.00% (Fig. 2). Group B patients, who continued to receive usual care and did not receive the I-MUR intervention until after the ACT score was obtained at T3, also had a statistically significant increase in ACT score (*p* < 0.01); 25/283 (9.90%) with poor or partial control achieved good control; the median ACT score increased from 18 to 19 (Table 2) and the proportion of patients with controlled asthma increased from 39.30 to 45.20% (Fig. 2). However, as the median ACT score in Group B at T3 was still below the threshold for good control (ACT ≥20), this may not be regarded as a clinically relevant change. The proportional increase in asthma control from T0 to T3 was 21% in Group A and 15% in Group B, the RR was 2, the RRR was 1.02 (95% CI 2.21–0.28), the ARR was 0.10 (95% CI 0.17–0.03) and the NNT was 10 (95% CI 6–28). At T6, asthma control improved further in both groups. The increase in Group B patients, 3 months after receiving the I-MUR intervention, was both statistically significant (*p* < 0.01) and clinically significant median ACT score increasing from 19 (not controlled) to 20 (controlled) (Table 2). The proportion of Group B patients controlled increased again at T9, although the median ACT score remained the same (*p* = 0.05). Patients in Group A continued to show improvements in ACT score both at T6 (ACT = 21) and T9 (ACT = 22), but only the improvement at T9 was statistically significant (*p* < 0.01).

**Table 2:** Median ACT scores plus inter quartile range (IQR) for Group A and Group B using both PP and ITT analyses

		T0	T3	T6	T9
PP	GA	19 (15–23)	20.5 (17–23)	21 (17–24)	22 (18–24)
	GB	18 (14–22)	19 (15–22)	20 (16–22.75)	20 (16–23)
ITT	GA	19 (14.25–23)	20 (16–23)	21 (16–23)	21 (17–24)
	GB	17 (17–21)	18 (14–21)	19 (15–22)	20 (15–22)

Intervention was delivered immediately after ACT at T0 in Group A and immediately after ACT at T3 in Group B, thus T6 scores are 6- and 3-months post-intervention for groups A and B respectively; T9 scores are 9- and 6-months post-intervention for groups A and B

Using this approach, the proportion of patients in both groups with controlled asthma continued to improve at each time point, but the median ACT score only reached a value indicating controlled asthma in both groups following the I-MUR intervention. The increase was greater in group B (29.00%) compared to group A (21.00%) (*p* < 0.01). From T0 to T9 the percentage increase in controlled patients was 40.20% and 45.00% respectively in Groups A and B. Thus asthma control was not only sustained but continued to increase throughout the study (Fig. 2).

Including all patients in an ITT analysis showed an even greater difference between the groups at baseline in the proportion with asthma control (ACT score ≥20) and that the median ACT score did not reach ≥20 for patients in Group B until T9, whereas patients in Group A achieved this at T3. However, as with the per protocol analysis, ACT score showed an overall increase in both groups at each time point (*p* < 0.01; Friedman’s ANOVA). The result of the GEE analysis applied to PP showed that the I-MUR intervention resulted in improved asthma control (ACT scores at T0 versus ACT scores at T3): patients receiving the intervention were 1.8 times more likely to improve from not controlled (ACT ≤ 19) to controlled (ACT ≥ 20) than control patients (odds ratio (OR) 1.76, 95% CI 1.33–2.33); the ICC was 0.07 (95% CI 0.008–0.168). A similar result was obtained also when a more stringent and conservative approach was used during the ITT analysis (OR 1.70, 95% CI 1.36–2.13).

### Secondary outcomes

#### Number of active ingredients

Data on asthma-related medicines regularly used by patients were gathered during the I-MUR intervention, at T0 for group A and at T3 for group B. There were no significant statistical differences between the two groups. The total number of active ingredients recorded by pharmacists as being used in 812 patients was 4310, median 5 per patient, range 0 - >20. After the I-MUR intervention, the total number of active ingredients was 3970 (*n* = 759), median 4, range 1 – 20, a reduction of *n* = 340 or 7.90% (*p* < 0.01). The reduction was maintained in both groups 6 months after the intervention (Table 3).

**Table 3:** Changes in the number of active ingredients used by patients before and after the I-MUR intervention

	Before I-MUR	3 months after I-MUR	6 months after I-MUR
GA	5 (3–7)	4 (3–7)	4 (3–7)
GB	5 (3–7)	4 (2–7)	4 (3–7)

Values are presented as median (IQR)

#### Adherence

**Fig. 3:**
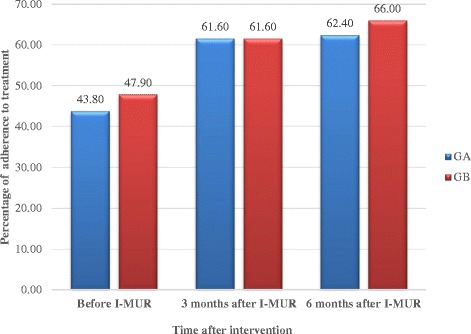
Patients’ self-reported adherence to medications before and after I-MUR

Self-reported adherence was recorded in 802 patients on three occasions during the study, using questions adapted from Morisky scale. Significant (*p* < 0.01) improvements were found in both groups at both 3 and 6 months post intervention, compared to before the I-MUR. The greatest improvement in adherence occurred at 3 months post-intervention, with the improvement sustained at 6 months (Fig. 3).

**Fig. 4:**
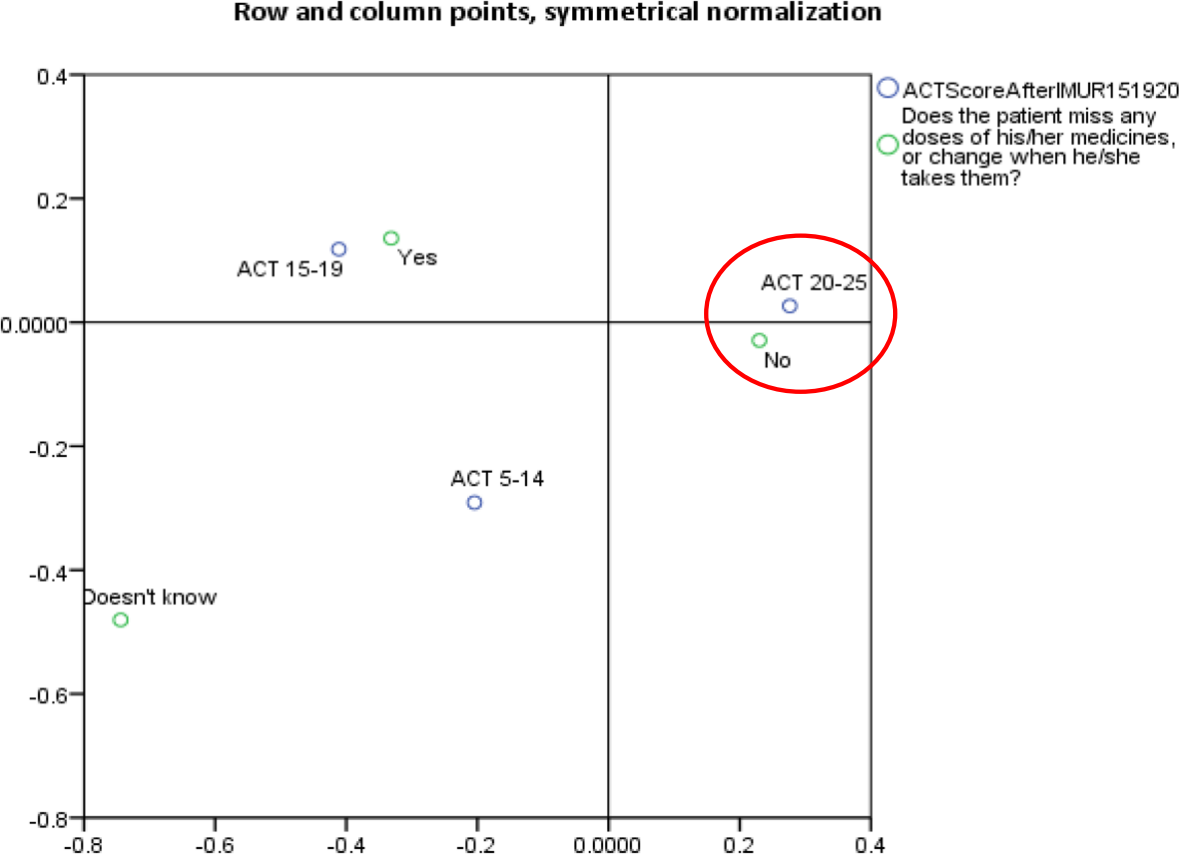
Relationship between asthma control and adherence to treatment after I-MUR

Correspondence analysis demonstrated that the patients who achieved asthma control (ACT 20–25) were those who did not miss or change their medication (Fig. 4).

#### Cost effectiveness analysis

At 3-months follow-up, only the difference in costs (from the public healthcare and society perspectives) were significant at the 0.01 level (Table 4). The bootstrap method was therefore employed to evaluate uncertainty surrounding cost-effectiveness estimates.

**Table 4:** Costs and QALY estimates for the three scenarios considered in the cost-utility analysis

	Intervention (*N* = 600)		Control (*N* = 663)		*p*-value
	Mean	Sd	Mean	Sd	*P*
Scenario 1: 3 months					
Difference in yearly patient costs between T3 and T0 (public healthcare perspective, [13] Euros 2015)	−122.63	747.03	−113.29	828.04	0.01
Difference in yearly patient costs between T3 and T0 (societal perspective, [6] Euros 2015)	−95.17	660.94	−95.82	741.32	0.01
Difference^1^ in QALYs between T3 and T0 [39]	0.02	0.10	0.01	0.10	0.86
Scenario 2: 6 months					
Difference in yearly patient costs between T6 and T0 (public healthcare, [13] Euros 2015)	−154.84	846.47	−113.29	828.04	0.01
Difference in yearly patient costs between T6 and T0 (societal perspective, [6] Euros 2015)	−115.93	741.45	−95.82	741.32	0.01
Difference in QALY s between T6 and T0 [39]	0.03	0.12	0.01	0.10	0.01
Scenario 3: 9 months					
Difference in yearly patient costs between T9 and T0 (public healthcare perspective, [13] Euros 2015)	−207.04	828.88	−113.29	828.04	0.01
Difference in yearly patient costs between T9 and T0 (societal perspective [6] Euros 2015)	−158.56	729.97	−96.12	741.37	0.01
Difference in QALYs between T9 and T0 [39]	0.04	0.11	0.01	0.10	0.01

**Fig. 5:**
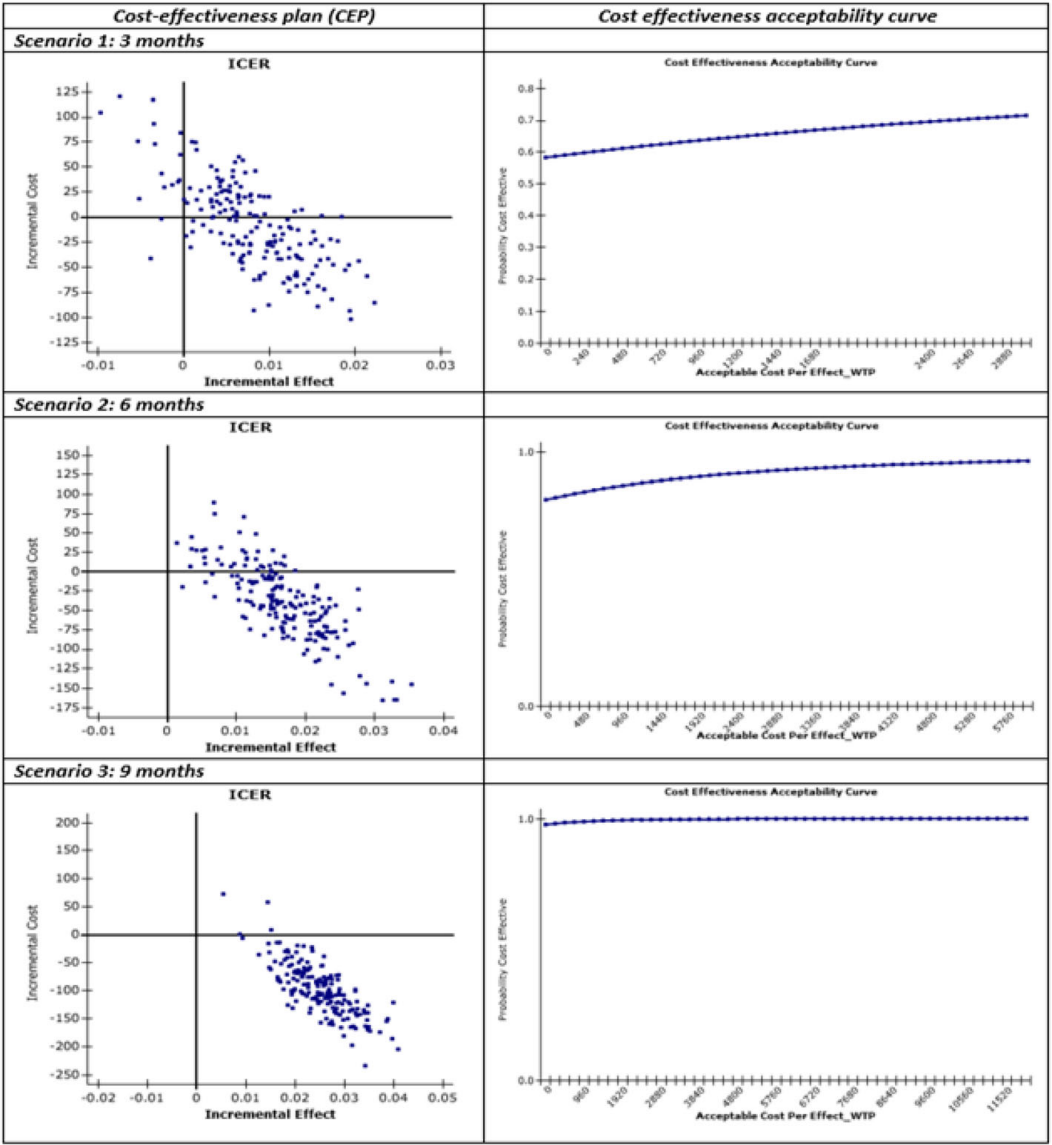
Cost Utility analysis (Italian public healthcare perspective, Euros 2015)

**Fig. 6:**
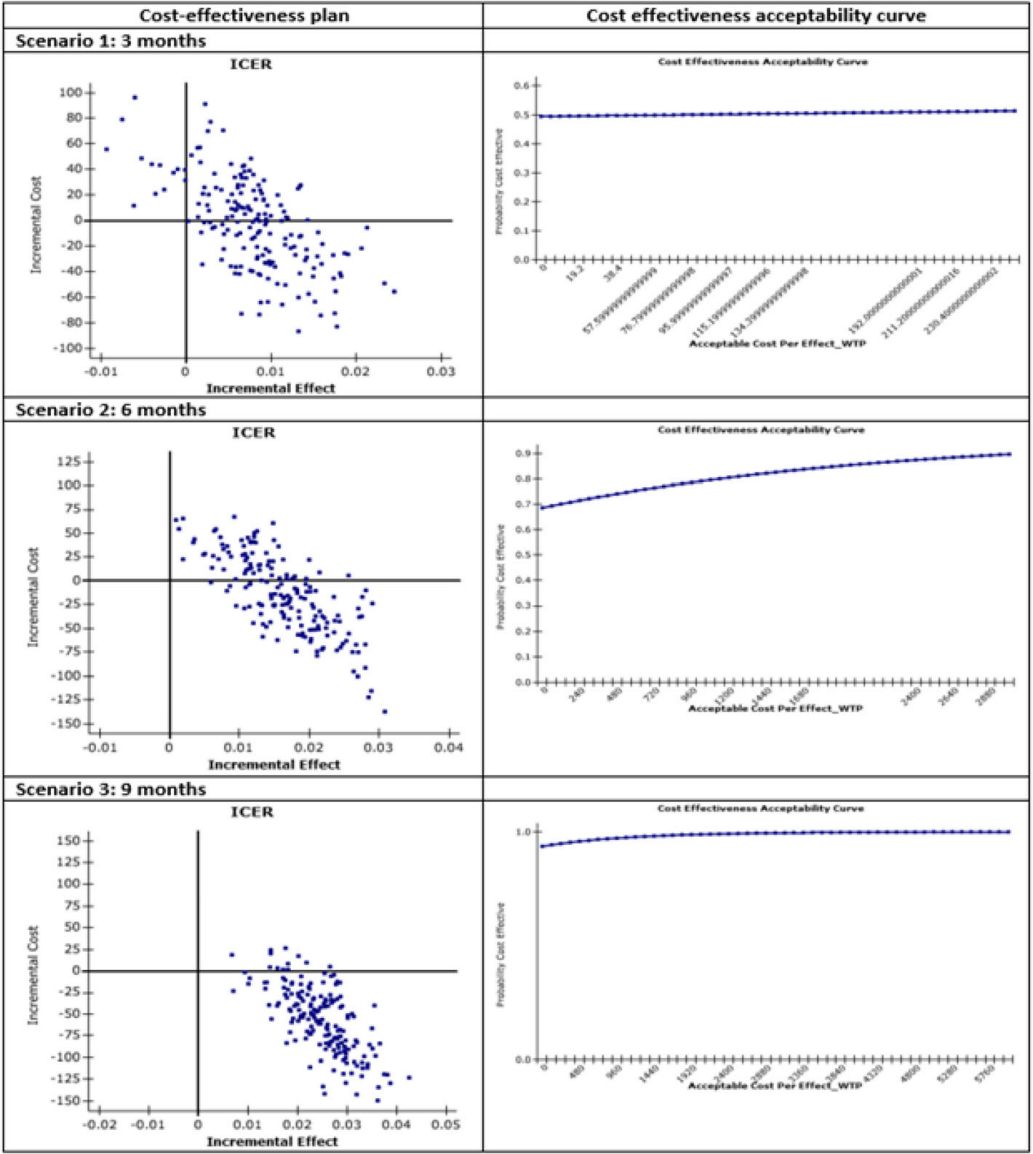
Cost utitlity analysis (society perspective, Euros 2015)

In the economic analysis we have used mean rather than median values, as this is more informative for planners of healthcare services. As the patient cost data were skewed, the confidence limits around the means were generated using non-parametric bootstrapping techniques. The incremental cost-effectiveness plane (CEP) showed 60.50% of the plots falling into the south-east quadrant, indicating that I-MUR service dominated the usual care option (less costly and more effective). Considering a WTP threshold of €30,000 per additional QALY gained, the probability that I-MUR was more cost-effective is 71.50%. For the secondary analysis, the incremental CEP showed about half of the plots falling into the south-east quadrant. The probability that I-MUR care was more cost-effective was 51.50% (Figs. 5 and 6). At 6 months, both the difference in costs and utility were significant at 0.01 level. The majority of plots in the cost-effectiveness scatter fell in the south-east quadrant (between 82.70 and 69.60%; public healthcare and society perspectives, respectively) and indicated that usual care was dominated by I-MUR service. The probability that I-MUR care was more cost-effective varied between 93.00 and 89.50%, respectively (Figs. 5 and 6).

At 9 months, both the difference in costs and utility were significant at 0.01 level. The large majority of plots in the cost-effectiveness scatter fell in the south-east quadrant (between 98.10 and 93.60%; public healthcare and society perspectives, respectively) and indicated that usual care was dominated by I-MUR service. The probability of I-MUR being cost-effective was 100% (Figs. 5 and 6). Overall the probability of I-MUR care being cost-effective doubled across time (from 51.50–71.50% at 3 months up to 100% at 9 months).

## Discussion

### Main findings

The effectiveness analysis showed evidence for the superiority of I-MUR service compared with usual care for patients with asthma in terms of the primary outcome of ACT score and all of the secondary outcomes. There was a statistically significant improvement in the proportion of patients with controlled asthma in both groups over the 9 months of follow-up and the median ACT score moved from partial to good control after the I-MUR intervention, at T3 in Group A and T6 in Group B. The NNT was 10, thus indicating a potentially useful intervention. Relatively few studies of pharmacist interventions report NNTs, however of those which do, values are similar to that found here [46, 47].

The I-MUR intervention was shown to reduce the number of active ingredients being used and to improve patients’ adherence to their medications. In addition, a clear link was demonstrated between adherence and ACT score, suggesting that this may be one mechanism whereby asthma control was improved. Adherence increased overall by 35.4% 3 months post-intervention and by 40.0% at 6 months. The types of pharmaceutical care issues identified by the pharmacists during the I-MUR were primarily related to the need for education, monitoring and potentially ineffective therapy. Hence the provision of advice on use of medicines and how to optimise their effectiveness, together with frequent monitoring is likely to be the mechanism whereby adherence and thus asthma control were improved. The continuous improvement in both asthma control and adherence in both groups suggests that regular contact with the pharmacist and completion of the ACT score was in itself an important factor.

Cost-effectiveness analysis supported the hypothesis that I-MUR is more cost effective than usual care and the probability of I-MUR being cost-effective doubled across time (from 51.50% at 3 months to 100% at 9 months). Moreover it was demonstrated that the key factor influencing asthma control was adherence, which pharmacists are well placed to influence, and the resultant improvements in both were sustained over at least 6 months (9 months in Group A).

### Comparison with other studies

Similar results have been found in RCTs of community pharmacists’ intervention in asthma in other countries, across Europe [27, 28], Australia [26] and North America [22], with the largest studies to date being conducted in Australia, Belgium and Spain [26–28]. One Australian study [48] showed both improved adherence and improved asthma control which was sustained for 12 months. The Belgian study [27] assessed adherence through prescription refill rates rather than patient completed questions, but also assessed patient knowledge about asthma medication, but showed no change in asthma control overall, only in a sub-group of patients with uncontrolled asthma. While the Spanish study [28] showed evidence of benefit, the work was funded by a pharmaceutical company and one of the patients’ inclusion criteria was the use of Budesonide/Formoterol, a medication produced by the same company.

Other studies have also shown potential benefits of pharmacist intervention in asthma, although many were small and some were uncontrolled [29]. The English MUR service, on which this intervention was based, has relatively little evidence to support its effectiveness [49] or cost effectiveness [50]. However one small, uncontrolled study has shown that the proportion of patients with controlled asthma rose from 41 to 55% after receiving an MUR [51]. A systematic review of fee-for-service pharmacist-led medication reviews in general showed that this type of service improves adherence and clinical markers in a range of conditions [52]. It is essential that future studies should incorporate both clinical outcomes and economic evaluations, to enable governments and other commissioners to assess the potential benefits of such services.

### Strengths and limitations

This is the first study in Italy to evaluate a community pharmacist-based intervention, and, to the best of our knowledge, it appears to be the largest RCT of a community pharmacy intervention in asthma conducted in any country in terms of the numbers of pharmacists and patients. Further strengths are that it adopted the ACT score as a measure of asthma control, as recommended by GINA, which allowed measurement of both effectiveness and cost-effectiveness (by translating ATC scores into QALYs) of the novel I-MUR intervention. The large number of pharmacists involved throughout the country supports the generalisability of the results at national level.

Standard practice in community pharmacy in Italy involves little clinical input, being mainly a supply function, therefore the patients were unused to a pharmacist taking an interest in their clinical status and providing information about optimising medicines use, which may have contributed to the effectiveness of the intervention. The pharmacists providing the I-MUR were responsible for both selecting patients and entering the data collected onto the electronic template, possible sources of bias. The population overall included more females than males (*p* < 0.01) and, more importantly, the proportion of patients not controlled differed between groups A and B, with median ACT scores at baseline of 19 and 18. The assessment of adherence did not use a validated tool, but instead used only two patient-reported questions, in the interests of brevity, which were similar to the questions used in the English MUR template. Also the initial follow-up period before the usual care group received the intervention was only 3 months. This meant that we had to assume no change in ACT control from T3 onwards for the economic analysis. In addition it was necessary to use secondary data rather than primary data collection on cost and utility, which were derived from different sources and were old estimates [6, 13], actualised at 2015 [45].

## Conclusion

This study demonstrates that the I-MUR service, which is the first cognitive pharmaceutical service to be delivered in Italy, was both effective and cost-effective. The Italian Government/Ministry of Health have since promoted a change of community pharmacy practice, with the I-MUR being the first nationally funded cognitive pharmaceutical service in Italy [53]. The work has therefore supported a significant cultural shift in Italian community pharmacy practice, promoting the change from a mainly logistic to a more patient-centred and clinically-oriented role of the community pharmacist in delivery of health care. Consideration is being given to extending the service to other respiratory conditions, but the successful service model applied here could enable community pharmacists in Italy to support the care of patients with a wide range of long-term conditions in the future. Moreover, it adds to the evidence base world-wide demonstrating the potential effectiveness and cost-effectiveness of community pharmacy medication reviews.

## Abbreviations

95% CI: 95% Confidence interval
ACT: Asthma control test
AHRQ: Agency for healthcare research and quality
ARR: Absolute risk reduction
CA: Correspondence analysis
CEAC: Cost-effectiveness acceptability curve
CEP: Cost-effectiveness plane
DALYs: Disability-adjusted life years
DE: Design effect
ESS: Effective sample size
EuroQol-5D: Survey instrument for describing health-related quality of life
FOFI: Italian Pharmacists’ Federation/Federazione Ordini Farmacisti Italiani
GEE: General estimating equations
GINA: Global initiative for asthma
IBM: International business machine
ICC: Intra-cluster correlation coefficient
ICER: Incremental cost-effectiveness ratio
I-MUR: Italian medicines use review
ITT: Intention to treat
LOCF: Last observation carried forward
m: Number of units (patients) per cluster
MMAS8: Eight item morisky medication adherence scale
MUR: Medicines use review
NNT: Number needed to treat
OR: Odds ratio
PCIs: Pharmaceutical care issues
PI: Principal investigator
PP: Per protocol
QALY: Quality-adjusted life-year
RCT: Randomised controlled trial
RR: Relative risk
RRR: Relative risk reduction
SIMG: Italian Society of General Medicine/Societá Italiana di Medicina Generale
SPSS: Statistical package for the social sciences
STATA: Statistics data analysis
T0: Time zero
T3: Time at three months
T6: Time at six months
T9: Time at nine months
WTP: Willingness-to-pay
